# Efficacy of cervical mobilization with post-isometric relaxation in managing mechanical neck pain, ROM, and functional limitations associated with myofascial trigger points

**DOI:** 10.1097/MD.0000000000036710

**Published:** 2023-12-29

**Authors:** Hussain Saleh Ghulam, Raee Saeed Alqhtani, Adel Alshahrani, Hashim Ahmed, Abdur Raheem Khan, Ashfaque Khan

**Affiliations:** a Department of Medical Rehabilitation Sciences, College of Applied Medical Sciences, Najran University, Najran, Saudi Arabia; b Department of Physiotherapy, Integral University, Lucknow, UP, India.

**Keywords:** functional disability, muscle tenderness, myofascial trigger points pain, neck pain

## Abstract

**Background:**

Sedentary lifestyle, age-related degenerative changes or traumatic injuries leads to cervical spine structural mal-alignment, which results in neck pain and other symptoms. Various therapeutic exercises and manual techniques have been proven to be beneficial in terms of managing these symptoms. This study aimed to determine the combined effects of cervical mobilization and post-isometric relaxation (PIR) technique on managing neck pain, cervical side flexion range of motion, and functional limitation in participants with mechanical neck pain linked with myofascial trigger points.

**Methods:**

This study followed a 2-arm, parallel-group, pretest-posttest randomized comparative design. Thirty participants with mechanical neck pain associated with myofascial trigger points aged 30.87 ± 4.45 years were randomly allocated to Groups 1 and 2. Group 1 received conventional intervention, PIR, and cervical mobilization techniques while Group 2 received conventional intervention and PIR technique only. Neck pain, muscle tenderness, cervical range of motion, and functional limitations were assessed using a visual analog scale, pressure pain threshold (PPT), goniometer, and neck disability index (NDI) questionnaire, respectively at baseline on day 1 and post-intervention on day 7, 14, and 21. Wilcoxon signed-rank test and the Mann–Whitney U test evaluated within-group and between-group analyses, respectively. Statistical significance was established at a 95% confidence interval, indicated by *P* < .05.

**Results:**

Significant differences (95% confidence interval [CI], *P* < .05) were observed within each group for all the outcomes scores when compared to the baselines across multiple time points. Significant variations were observed between the groups when comparing visual analog scale and NDI scores at weeks 1, 2, and 3 post-interventions. In contrast, insignificant differences (95% CI, *P* > .05) were observed for side flexion range of motion and PPT compared at weeks 1, 2, and 3 post-interventions except for PPT at week 3 post-intervention (95% CI, *P* < .05). Additionally, Cohen *d* test revealed the superiority of group 1 over group 2 in reducing pain and functional limitations and improving cervical side flexion range of motion and PPT.

**Conclusion:**

The combination of cervical mobilization and Post-isometric relaxation techniques was discovered to effectively alleviate neck pain and enhance functional abilities when contrasted with the application of post-isometric relaxation alone in patients with mechanical neck pain linked with myofascial trigger points.

## 1. Introduction

The structural arrangement of the cervical spine makes it susceptible to mechanical changes,^[1]^ often triggered by degenerative shifts and improper posture,^[1]^ resulting in prevalent neck discomfort, with a 54% prevalence rate over 6 months.^[1]^ Typically, individuals experiencing neck pain turn to physiotherapy during the chronic phase, impeding their daily activities. However, a comprehensive understanding of the exact patho-mechanism is still lacking.^[2]^ Globally, approximately 37% of the population encounters mechanical neck pain at some point in their lives,^[3]^ marked by its gradual onset.^[4]^ A multitude of factors, including inadequate posture, neck strain, psychological elements, and specific activities, contribute to the emergence of neck pain.^[5]^ The limitation in neck mobility can be attributed to the tightening of the upper Trapezius and Levator scapulae muscles.^[6]^

Myofascial trigger points (MTrPs) play a significant role in the context of mechanical neck pain.^[7]^ As per Simons et al^[8]^, MTrPs are described as sensitive points within tense muscle bands, which trigger pain upon compression, contraction, or extension, often leading to pain referred to a distant area. These trigger points encompass various types that contribute to discomfort and impairment, predominantly classified as active and latent MTrPs. Active MTrPs are characterized by pain, tenderness, muscle weakness, and contraction. The diagnosis involves patient-reported pain during muscle compression and a localized twitch response upon stimulation. Active MTrPs also induce referred motor sensations and heightened sympathetic activity, with tenderness in the pain referral zone during muscle compression. Conversely, latent MTrPs lack pain but display tenderness during palpation, sharing clinical indications with active MTrPs except for immediate pain.^[9]^

Numerous manual and electrotherapy options, including TPI,^[10]^ massage, ultrasound, and more, are employed to address myofascial trigger points. Yet, no definitive evidence supports a particular approach for managing MTrPs effectively.^[11]^

Manual therapists increasingly turn to post-isometric relaxation (PIR) to address tight muscles and impaired joint function in myofascial pain syndrome.^[12]^ PIR involves collaborative resistance from the patient and therapist. Lewit and Simons noted reduced myofascial pain after PIR therapy. PIR’s effectiveness arises from its multifaceted impact, including muscle elongation,^[13]^ strengthening,^[14]^ aiding lymphatic/venous drainage,^[15]^ and improving joint range of motion. PIR necessitates voluntary muscle contraction against therapist-applied resistance.^[8]^ Wilson et al^[16]^ reported reduced pain sensitivity with PIR in low-back pain. Conversely, Ptaszkowski et al^[17]^ discovered no extra benefits of PIR over Kinesio taping for heightened upper Trapezius muscle tone and pain.

Spinal manipulation is a commonly used therapy for neck disorders and is effective.^[18]^ Limited evidence supports spinal mobilization for trigger points functional disability reduction and spine mobility. Gross et al^[19]^ found comparable effects of cervical manipulation and mobilization on pain, function, and satisfaction.

Recent research demonstrates PIR’s effectiveness in myofascial pain, stiffness, and mobility management in mechanical neck pain patients.^[20,21]^ Earlier, mobilizations improved AROM and reduced stiffness in cervical pain individuals.^[22]^

Various studies have demonstrated the efficacy of single interventions like cervical mobilization and PIR in addressing neck pain, cervical range of motion (ROM), and functional limitations linked to mechanically induced pain from MTrPs.^[18–23]^ Limited studies have explored combined approaches, including manual techniques, deep friction massage, electrotherapy, or positional release, for managing MTrP-associated neck pain, ROM, and limitations.^[24–27]^ However, the synergistic impact of cervical mobilization (CM) and PIR techniques remains unexplored in this context. This study seeks to address this gap by investigating the effectiveness of combining cervical mobilization and post-isometric relaxation for managing mechanical neck pain associated with active upper Trapezius MTrPs. This study hypothesized that a combination of cervical mobilization and post-isometric relaxation technique would be more beneficial than the post-isometric relaxation technique alone to reduce pain, improve ROM, and reduce functional limitation in participants with mechanical neck pain linked with MTrPs.

## 2. Methods

### 2.1. Study design

This study was based on a double-blind, 2-arm parallel-group randomized comparative design with a pretest-posttest assessment in nature.

### 2.2. Study participants

Participants aged between 20 and 40 years with mechanical neck pain localized in the neck or scapular areas and 1 to 2 active MTrPs in the upper Trapezius muscle (unilateral) were included. Exclusions encompassed cases of fibromyalgia syndrome, cervical radiculopathy or myelopathy, prior cervical spine surgery, congenital/acquired postural deformity, active MTrPs in bilateral upper Trapezius muscles, and treatment for pain within 1 month before the study.

### 2.3. Study settings

Thirty participants with mechanical neck pain related to upper Trapezius trigger points were enrolled at the Physiotherapy department of Najran University. Detailed information on the study’s aims, methods, and procedures was provided through written materials, and written informed consent was obtained. Participants were randomly divided into 2 equal groups (Group 1 and Group 2) using a simple random sampling method (i.e., lottery method) by the investigator. Group 1 received interventions including hot packs, active stretching, isometric exercises, Post-isometric relaxation technique, and cervical mobilization, whereas Group 2 received the same interventions excluding cervical mobilization.

### 2.4. Sample size

A computer software, G*Power 3.1.9.4, applied a priori *t* test for the mean of 2 independent samples, obtaining the visual analog scale (VAS) scores to estimate the effective sample size of the study. To assess the intervention effect size, a pilot study was conducted on 12 individuals (6/group). Keeping a power at 0.80 (80%), α error probability 0.05, (95% confidence interval [CI], 2-tailed), mean ± standard deviation difference 1.05 ± 0.031, allocation ratio N2/N1 = 1, and effect size d = 1.239, a sample of 24 participants (12/group) was required to satisfy the power of the study sample. Assuming a 20% attrition of the participants, a total sample of thirty participants was estimated to conduct this study.

### 2.5. Ethical consideration

The Ethical Committee of Najran University (Reference No.444-42-20993-DS, dated October 27, 2022) approved this study. A trial registration for this study was completed online at ClinicalTrial.gov Protocol Registration System, under Trial ID: NCT05684809, dated March 23, 2023.

### 2.6. Study outcomes

Pain intensity was assessed using a VAS, which consisted of a straight line of 10 cm in length. This line was marked with 2 labels, specifically “no pain” and “worst possible pain”, positioned at opposite ends of the line. The participants are provided with instructions to create a vertical mark on the line to indicate their level of pain. This method is considered highly reliable and consistent for pain measurement.^[28]^ Cervical side flexion range of motion was measured using a cervical range of motion device. Participants, seated upright, laterally flexed their heads from a neutral position. The assessor took 2 readings, and the average was analyzed. Functional limitations due to pain were evaluated with the Neck Disability Index questionnaire. The neck disability index (NDI) questionnaire helps assess how neck pain affects daily life. It consists of questions about personal care, lifting objects, working, sleeping, and recreation. Each of the 10 items is scored from 0 to 5. The maximum score is, therefore, 50. Each section is scored on a 0 to 5 rating scale, in which zero means “No pain” and 5 means “Worst imaginable pain”. asked the participants to rate the difficulty they experienced for each activity using a scale from 0 to 50, indicating higher disability with greater scores.^[29]^ Baseline and post-intervention data (pressure pain threshold [PPT], VAS, cervical ROM, and NDI) were gathered on the 7th, 14th, and 21st days.

### 2.7. Measurements

To assess myofascial trigger point pain sensitivity, the pressure threshold meter (“WAGNER FORCE DIAL FDK 20”) was utilized.^[30,31]^ Trigger points in the neck were marked, with the lowest pressure pain threshold being designated as the primary 1. Participants reported changes in pressure sensation turning painful. The assessor recorded 3 measurements, with the average considered. A minimum of 1-minute intervals was maintained between measurements.^[30,31]^

### 2.8. Procedures

**Figure 1.**
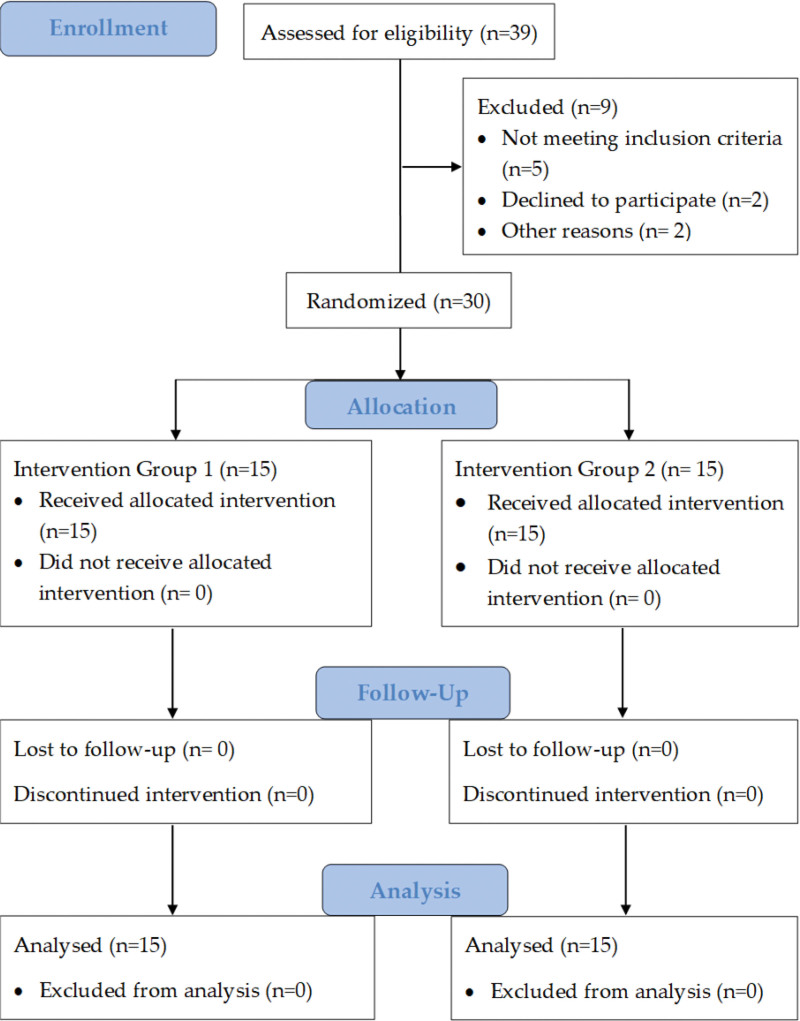
A CONSORT (2010) flow chart depicts the study procedures, including recruitment, randomization, allocation, follow-up, and analysis.

A CONSORT (2010) flow diagram presents the study’s procedures, including recruitment, randomization, allocation, follow-up, and analysis in Figure 1.

## 3. Interventions

Besides a conventional intervention for both groups, Group 1 received post post-isometric relaxation technique and cervical mobilization technique, and Group 2 received a post-isometric relaxation technique alone. The study duration was 3 weeks.

### 3.1. Conventional intervention

In conventional intervention, the patients received hot packs on the cervical paraspinal region, which included the upper trapezius muscle with MTrPs (75^0^C for 20 minutes).^[32]^ Active stretching was done (5 repetitions, 20 seconds hold, 10 seconds relaxation), and isometric neck exercises (10 repetitions, 2 sets, 5–10 seconds hold as pain allowed).^[33]^ Exercises were performed thrice daily for 21 days. Isometric exercises included neck rotation, flexion, extension, and lateral bending. The rest time period between 2 repetitions of each exercise was 30 to 60 seconds.

### 3.2. Post-isometric relaxation technique

For post-isometric relaxation, patients lay supine, neck laterally flexed, lengthening the upper Trapezius muscle. The therapist conducted a moderate isometric contraction (about 75% max) of the trapezius, held for 5 seconds, relaxed for 3 seconds, and then moved the cervical spine beyond the barrier.^[34]^ Each session repeated these 4 times. The participants practiced with a moderate isometric contraction of 75% max in a familiarization session conducted prior to the start of the intervention.

### 3.3. Cervical mobilization technique

In Cervical Mobilization, Grades used were dependent upon the chief complaint and desired goals. Grades I and II were employed when pain appeared prior to movement restriction. Grades III and IV were used when resistance to movement was encountered before pain.^[35]^ Patients assumed a prone position with foreheads on their hands and chins tucked. The therapist stood at their head, thumbs opposed and tips on the vertebra spinous process to be mobilized. Fingers supported the neck and head sides, enhancing thumb balance and stability. Extremely gentle pressure was then applied to produce a feeling of movement, the pressure was applied using the arms combined with the trunk, not by the action of intrinsic muscles. The direction of central pressure is directed towards the facet joint plane (treatment plane) downwards and forwards at a 45˚ angle. The treatment sessions were conducted every other day (3 sessions per week) over a span of 3 weeks.

### 3.4. Statistical analysis

IBM SPSS version 26 (Statistical Package for the Social Sciences) software was employed for data analysis [IBM Corp., 2019. IBM SPSS Statistics for Windows, Version 26.0. Armonk, NY: IBM Corp]. Normality was assessed using the Shapiro–Wilk test. Wilcoxon signed-rank test and the Mann–Whitney *U* test were utilized for within-group and between-group effects evaluation. A Cohen *d* test was utilized to compare the intervention effect size on the outcome variables to reveal the superiority between the groups. A significance level of *P* < .05 was maintained for all analyses, with a 95% confidence interval.

## 4. Results

Thirty out of thirty-nine participants were screened, recruited, and randomly allocated to Group 1 and Group 2. Nine participants were excluded from the study. Out of 9 excluded participants, 5 did not match the study eligibility criteria, 2 declined to participate without any reason, and 2 participants did not participate due to some valid reason (Fig. 1). A Shapiro–Wilk test of normality revealed that the participants were normally distributed (95% CI, *P* > .05) in both groups by demographic characteristics, such as age (years), height (cm), weight (kg), and BMI (kg/m^2^), and baseline values for all the outcomes except VAS and NDI (*P* < .05), as presented in Table 1.

**Table 1:** Showing demographic **c**haracteristics of the participants, baseline scores for the outcomes, and test for normality using the Shapiro–Wilk test (95% CI for mean).

Variables	Groups (n = 15/group)	Baseline (mean ± SD)	Shapiro–Wilk test of normality		
			Statistics	df	*P* value
Age (yr)	Group 1	30.60 ± 4.672	.902	15	.104
	Group 2	31.13 ± 4.224	.949	15	.504
Weight (kg)	Group 1	57.53 ± 5.617	.934	15	.309
	Group 2	58.67 ± 6.831	.958	15	.665
Height (cm)	Group 1	167.16 ± 7.933	.896	15	.083
	Group 2	165.75 ± 7.099	.959	15	.679
BMI (Kg/m^2^)	Group 1	20.55 ± 1.362	.928	15	.254
	Group 2	21.13 ± 1.202	.897	15	.085
VAS (cm)	Group 1	6.53 ± 0.516	.643	15	.001*
	Group 2	6.60 ± 0.507	.630	15	.001*
NDI	Group 1	18.93 ± 0.961	.868	15	.032*
	Group 2	19.40 ± 1.183	.897	15	.086
SF-ROM (^0^)	Group 1	36.00 ± 1.512	.921	15	.200
	Group 2	35.40 ± 1.595	.913	15	.148
PPT (Nm^-2^)	Group 1	1.75 ± 0.465	.944	15	.441
	Group 2	1.74 ± 0.396	.925	15	.233

BMI = body mass index, CI = confidence interval, df = Degree of freedom, NDI = Neck disability index, SF-ROM = cervical side flexion (unaffected side) Range of Motion, PIR = post-isometric relaxation, PPT = pain pressure threshold, VAS = visual analog scale.

* Significant value if *P* < .05.

## 5. Within-group analysis

The Wilcoxon signed-rank test revealed significant differences (95% CI, *P* < .05) within each group for the outcomes scores of VAS, NDI, side flexion range of motion (SF-ROM), and PPT when compared the baselines across multiple time points scores, as presented in Tables 2 and 3.

**Table 2:** A within-group comparison of mean outcomes scores of VAS, NDI, SF-ROM, and PPT in Group 1, using a Wilcoxon signed-rank test (two-tailed, N = 15).

Variables	Wilcoxon signed-rank test (two-tailed, N = 15)			
	Mean rank	Sum of rank	Z statistics	*P* value
VAS1–VAS0	8.00	120.00	−3.535	.001*
VAS2–VAS0	8.00	120.00	−3.472	.001*
VAS3–VAS0	8.00	120.00	−3.624	.001*
VAS3–VAS1	8.11	113.50	−3.234	.001*
NDI1–NDI0	8.00	120.00	−3.520	.001*
NDI2–NDI0	8.00	120.00	−3.461	.001*
NDI3–NDI0	8.00	120.00	−3.477	.001*
NDI3–NDI1	6.00	66.00	−3.071	.002*
SF1–SF0	.00	.00	−3.578	.001*
SF-ROM2-SF-ROM0	.00	.00	−3.508	.001*
SF-ROM3-SF-ROM0	.00	.00	−3.472	.001*
SF-ROM3-SF-ROM1	.00	.00	−3.213	.001*
PPT1–PPT0	.00	.00	−3.438	.001*
PPT2–PPT0	.00	.00	−3.436	.001*
PPT3–PPT0	.00	.00	−3.418	.001*
PPT3–PPT1	.00	.00	−3.423	.001*

NDI = neck disability index, SF-ROM = cervical side flexion (unaffected side) range of motion, PPT = pain pressure threshold, VAS = visual analog scale, Z = Z statistics.

* Significant value if *P* < .05.

**Table 3:** A within-group comparison of mean outcomes scores of VAS, NDI, SF-ROM, and PPT in Group 2, using a Wilcoxon signed-rank test (two-tailed N = 15).

Variables	Mean rank	Sum of rank	Z statistics	*P* Value
VAS1–VAS0	7.50	105.00	−3.638	.001*
VAS2–VAS0	7.50	105.00	−3.416	.001*
VAS3–VAS0	8.00	120.00	−3.473	.001*
VAS3–VAS1	7.00	91.00	−3.272	.001*
NDI1–NDI0	8.00	120.00	−3.690	.000*
NDI2–NDI0	8.00	120.00	−3.508	.001*
NDI3–NDI0	8.00	120.00	−3.457	.001*
NDI3–NDI1	7.50	105.00	−3.336	.001*
SF-ROM1-SF-ROM0	.00	.00	−3.690	.001*
SF-ROM2-SF-ROM0	.00	.00	−3.487	.001*
SF-ROM3-SF-ROM0	.00	.00	−3.475	.001*
SF-ROM3-SF-ROM1	.00	.00	−3.127	.002*
PPT1–PPT0	.00	.00	−3.420	.001*
PPT2–PPT0	.00	.00	−3.415	.001*
PPT3–PPT0	.00	.00	−3.412	.001*
PPT3–PPT1	.00	.00	−3.415	.001*

NDI = neck disability index, SF-ROM = cervical side flexion (unaffected side) range of motion, PPT = pain pressure threshold, VAS = visual analog scale, Z = Z statistics.

* Significant value if *P* < .05.

## 6. Between-group analysis

The Mann–Whitney U test revealed that all the outcomes were well matched (95% CI, *P* > .05) between the groups at the baseline scores, indicating a successful randomization procedure having identical groups at the study beginning. Significant variations were observed between the groups when comparing the outcomes scores of the VAS and NDI at weeks 1, 2, and 3 post-interventions. In contrast, insignificant differences (95% CI, *P* > .05) were observed for the outcomes scores of SF-ROM and PPT compared at weeks 1, 2, and 3 post-intervention except for PPT at week 3 post-intervention (95% CI, *P* < .05), as presented in Table 4.

**Table 4:** Between-group comparison of mean outcomes scores of VAS, NDI, SF-ROM, and PPT at weeks 0, 1, 2, and 3 post-intervention using the Man–Whitney U test (N = 30).

Variables	Groups (mean ± SD) (N = 15/group)		Man–Whitney *U* test (95% CI, two-tailed)		Cohen *d*
	Group 1	Group 2	Z statistics	*P* value	*d* value
VAS0	6.53 ± 0.52	6.60 ± 0.51	−.362	.717	0.136
VAS1	3.93 ± 0.88	5.60 ± 0.51	−4.134	.001*	2.322
VAS2	3.33 ± 0.90	5.06 ± 0.88	−3.912	.001*	1.943
VAS3	2.86 ± 0.83	4.26 ± 0.96	−3.557	.001*	1.560
NDI0	18.93 ± 0.96	19.40 ± 1.18	−.952	.341	0.436
NDI1	15.53 ± 0.99	18.26 ± 1.28	−4.385	.001*	2.385
NDI2	14.93 ± 0.96	16.86 ± 1.06	−3.858	.001*	1.909
NDI3	14.60 ± 0.83	16.20 ± 0.86	−3.841	.001*	2.626
SF-ROM0	36.00 ± 1.51	35.40 ± 1.59	−1.079	.281	0.387
SF-ROM1	37.73 ± 1.53	37.27 ± 1.58	−.889	.374	0.296
SF-ROM2	38.46 ± 1.46	37.80 ± 1.52	−1.298	.194	0.443
SF-ROM3	38.80 ± 1.47	38.13 ± 1.64	−1.252	.211	0.436
PPT0	1.75 ± 0.47	1.74 ± 0.40	−.104	.917	0.023
PPT1	2.19 ± 0.52	2.38 ± 0.42	−.978	.328	0.402
PPT2	2.95 ± 0.52	2.90 ± 0.38	−.687	.492	0.110
PPT3	3.28 ± 0.50	2.94 ± 0.27	−2.331	.020*	0.846

CI = confidence interval, NDI = neck disability index, SF-ROM = cervical side flexion (unaffected side) range of motion; PPT = pain pressure threshold, VAS = visual analog scale, Z = Z statistics.

* Significant value if *P* < .05.

Additionally, Cohen *d* test compared the intervention effect size on the outcome variables between the groups. It revealed the superiority of group 1 over group 2 in reducing pain and functional limitations and improving side flexion muscle endurance and pain pressure threshold, in patients with mechanical neck pain.

## 7. Discussion

The investigation was conducted to assess the effectiveness of combining cervical mobilization and post-isometric relaxation techniques in addressing pain intensity, pressure pain threshold, cervical side flexion range of motion, and functional limitations in individuals afflicted by mechanical neck pain linked to upper trapezius active myofascial trigger points. The outcome scores of pain intensity, muscle tenderness, cervical SF-ROM, and functional limitations were measured using VAS, PPT, cervical range of motion, and NDI, respectively. A Shapiro–Wilk test of normality revealed that the participants were normally distributed to their respective groups regarding demographic characteristics, such as age, height, weight, and BMI; and for all the study’s outcomes scores at baseline, except for the outcomes scores of VAS in Group 1 and Group 2 and PPT in Group 1 only.

Thirty participants, 15 in each group were randomly distributed to their respective group to counter the invariability effect of the sample. In addition, this study utilized nonparametric tests, such as the Wilcoxon Signed-Rank and Mann–Whitney *U* tests that are suitable for analyzing paired data within-group (Tables 2. and 3.) and between-group (Table 4.) when the assumptions of parametric tests are not met and to ensures robust and reliable results even with small sample sizes and non-normally distributed data (Table 1.).

A Wilcoxon signed-rank test revealed the overall, significant differences in all variables across different time points (*P* < .001), indicating strong evidence against the null hypothesis. The mean ranks and sum of ranks for each variable indicate the average ranking and total sum of ranks across the sample. The Z statistics values were negative for all variables, indicating a decrease in the respective measures from the baseline within the sample. This suggests the effectiveness of the combined (PIR + CM) or single (PIR) interventions in producing significant improvements (*P* < .05) in pain, disability, range of motion, and pressure pain threshold.

The findings of this study are consistent with previous research,^[36,37]^ which also reported significant improvements in similar variables following similar interventions. The study conducted by Hashim et al (2020) revealed that both Post-Isometric Relaxation and Laser Therapy interventions demonstrated efficacy in decreasing pain pressure threshold and pain intensity among patients with muscle trigger point discomfort. In a study conducted by Junaid M et al^[38]^ (2020), it was shown that the implementation of the post-isometric relaxation approach demonstrated enhanced and expedited outcomes in reducing pain and impairment, as well as enhancing mobility in patients with acute mechanical neck discomfort.

The Mann–Whitney U test revealed the nonsignificant differences between the groups for all the outcome scores at baseline, indicating a successful randomization procedure in avoiding unmatched group comparison at starting points.

Regarding post-intervention results, notable distinctions emerged between the 2 groups concerning pain intensity as evaluated by the Visual Analog Scale and quality of life as evaluated by the Neck Disability Index scores during weeks 1, 2, and 3. This suggests that the combination of cervical mobilization and post-isometric relaxation technique resulted in greater improvements in pain intensity and quality of life compared to post-isometric relaxation alone. These findings align with previous studies supporting the efficacy of combined interventions in reducing pain and improving quality of life in patients with neck pain and myofascial trigger points.^[25,26,39]^

Conversely, no significant variations were detected between the groups in cervical SF-ROM and PPT scores at weeks 1, 2, and 3 post-interventions, except for PPT at week 3. These results suggest that adding CM to PIR did not lead to significant variation in cervical SF-ROM and PPT scores compared to the PIR technique alone. It is worth noting that the lack of significant differences in cervical SF-ROM and most of the PPT measurements may be attributed to various factors, such as the specific characteristics of the study population, the duration and intensity of the intervention, and individual variability in response to treatment.^[40–43]^

The significant difference observed in PPT at week 3 post-intervention indicates that the combined intervention had a positive effect on the pain pressure threshold compared to PIR alone. This finding may suggest a delayed or cumulative response to the intervention, requiring further investigation to elucidate the underlying mechanisms and clinical implications.

Besides the noticeable intervention effects on the study’s outcomes, this study acknowledges its certain limitations. The study’s population size and specific characteristics may limit the generalizability of the findings. Additionally, the study duration and follow-up period might not have been sufficient to capture long-term effects or potential relapses. Furthermore, the inclusion of additional outcome measures, such as functional status and patient-reported outcomes, would have provided a more comprehensive evaluation of the treatment effects.

## 8. Conclusion

This study illustrated that the combination of cervical mobilization and post-isometric relaxation technique led to a significant reduction in pain intensity and functional limitations compared to post-isometric relaxation alone in participants with mechanical neck pain linked to myofascial trigger points. However, except for pain pressure threshold at week 3 post-intervention, no significant differences were evident between the groups in cervical side flexion range of motion and pain pressure threshold. These results contribute to the existing evidence supporting combined manual therapy interventions for myofascial-triggered neck pain management. Further research is needed to explore long-term effects, optimal treatment protocols, and underlying mechanisms.

## Acknowledgments

The authors are thankful to the Deanship of Scientific Research at Najran University for funding this work under the Research Priorities and Najran Research Funding Program (NU/NRP/MRC/12/5).

## Author contributions

**Conceptualization:** Hussain Saleh Ghulam, Raee Saeed Alqhtani, Hashim Ahmed, Adel Alshahrani, Abdur Raheem Khan and Ashfaque Khan.

**Data curation:** Raee Saeed Alqhtani, Hashim Ahmed, Abdur Raheem Khan, Abdur Raheem Khan.

**Formal analysis:** Raee Saeed Alqhtani, Hashim Ahmed.

**Funding acquisition:** Raee Saeed Alqhtani.

**Investigation:** Hashim Ahmed.

**Methodology:** Raee Saeed Alqhtani, Adel Alshahrani, Hashim Ahmed, Abdur Raheem Khan, Ashfaque Khan.

**Project administration:** Raee Saeed Alqhtani.

**Resources:** Adel Alshahrani, Hashim Ahmed.

**Software:** Hashim Ahmed.

**Supervision:** Hussain Saleh Ghulam, Raee Saeed Alqhtani.

**Validation:** Hussain Saleh Ghulam, Raee Saeed Alqhtani, Hashim Ahmed.

**Visualization:** Hussain Saleh Ghulam, Raee Saeed Alqhtani, Adel Alshahrani.

**Writing – original draft:** Hashim Ahmed.

**Writing – review & editing:** Raee Saeed Alqhtani, Adel Alshahrani, Hashim Ahmed, Abdur Raheem Khan, Ashfaque Khan.

## References

[1] Nagrale AV Glynn P Joshi A. The efficacy of an integrated neuromuscular inhibition technique on upper trapezius trigger points in subjects with non-specific neck pain: a randomized controlled trial. J Man Manip Ther. 2010;18:37–43. 21655422 10.1179/106698110X12595770849605 PMC3103119

[2] Childs JD Cleland JA Elliott JM. Neck pain: clinical practice guidelines linked to the international classification of functioning, disability, and health from the orthopaedic section of the American Physical Therapy Association. J Orthop Sports Phys Ther. 2008;38:A1–A34. 10.2519/jospt.2008.0303 18758050

[3] Ssavedra-Hernández M Castro-Sánchez AM Fernández-de-las-Peñas C. Predictors for identifying patients with mechanical neck pain who are likely to achieve short-term success with manipulative interventions directed at the cervical and thoracic spine. J Manipulative Physiol Ther. 2011;34:144–52. 21492749 10.1016/j.jmpt.2011.02.011

[4] Clair DA Edmondston SJ Allison GT. Physical therapy treatment dose for nontraumatic neck pain: a comparison between 2 patient groups. J Orthop Sports Phys Ther. 2006;36:867–75. 17154140 10.2519/jospt.2006.2299

[5] Heintz MM Hegedus EJ. Multimodal management of mechanical neck pain using a treatment based classification system. J Man Manip Ther. 2008;16:217–24. 19771194 10.1179/106698108790818260 PMC2716155

[6] Mahajan R Kataria C Bansal K. Comparative effectiveness of muscle energy technique and static stretching for treatment of subacute mechanical neck pain. Int J Health Rehabil Sci. 2012;1:16–21.

[7] Nachemson A Waddell G Norlund AI. Epidemiology of neck and low back pain neck and back pain, the scientific evidence of causes, diagnosis, and treatment. Philadelphia, PA: Lippincott Williams and Wilkins; 2000:165–87.

[8] Simons DG Travell J Simons LS. Myofascial Pain and Dysfunction: The Trigger Point Manual. 2nd ed. Baltimore, MD: Williams and Wilkins; 1999;1.

[9] Bron C Dommerholt J Stegenga B. High prevalence of shoulder girdle muscles with myofascial trigger points in patients with shoulder pain. BMC Musculoskelet Disord. 2011;12:139. 21711512 10.1186/1471-2474-12-139 PMC3146907

[10] Wong CS Wong SH. A new look at trigger point injections. Anesthesiol Res Pract. 2012;2012:492452. 21969825 10.1155/2012/492452 PMC3182370

[11] Hsueh TC Cheng PT Kuan TS. The immediate effectiveness of electrical nerve stimulation and electrical muscle stimulation on myofascial trigger points. Am J Phys Med Rehabil. 1997;76:471–6. 9431265 10.1097/00002060-199711000-00007

[12] Manheim C. The Myofascial Release Manual. 3rd ed. New Jersey, USA, Illus: Slack Inc; 2001;304:$45

[13] Ahmed H Miraj M Katyal S. Effect of muscle energy technique and static stretching on hamstring flexibility in healthy male subjects. Indian J Physiother Occup Ther. 2010;4:32–6.

[14] Ballantyne F Fryer G McLaughlin P. The effect of muscle energy technique on Hamstring extensibility: the mechanism of altered flexibility. J Osteopath Med. 2003;6:59–63.

[15] Nagarwal AK Zutshi K Ram CS. Improvement of hamstring flexibility: a comparison between two PNF stretching techniques. Int J Sports Sci Eng. 2010;4:25–33.

[16] Wilson E Payton O Donegan-Shoaf L. Muscle energy technique in patients with acute low back pain: a pilot clinical trial. J Orthop Sports Phys Ther. 2003;33:502–12. 14524509 10.2519/jospt.2003.33.9.502

[17] Ptaszkowski K Slupska L Paprocka-Borocwicz M. Comparison of the short-term outcomes after postisometric muscle relaxation or kinesio taping application for normalization of the upper trapezius muscle tone and the pain relief: a preliminary study. Evid Based Complement Alternat Med. 2015;2015:721938. 26347792 10.1155/2015/721938 PMC4549535

[18] Pickar J. Neurophysiological effects of spinal manipulation. Spine J. 2002;2:357–71. 14589467 10.1016/s1529-9430(02)00400-x

[19] Gross A Miller J D’Sylva J. Manipulation or mobilisation for neck pain. Cochrane Database Syst Rev. 2010. 10.1002/14651858.CD004249.pub3 20091561

[20] Chaitow L Judith WD. Clinical Application of Neuromuscular Technique. The Upper Body Churchill Livingstone. 2001:1.

[21] Simons DG. Diagnostic criteria of myofascial pain caused by trigger points. J Musculoskelet Pain. 1999;7:111–20.

[22] Tuttle N Barrett R Laakso L. Relation between changes in posteroanterior stiffness and active range of movement of the cervical spine following manual therapy treatment. Spine (Phila Pa 1976). 2008;33:E673–9. 18758348 10.1097/BRS.0b013e31817f93f9

[23] Saleem S Chaudhry SZ Sohail MU. Comparative effects of strain counterstrain and ischemic compression technique in patients with upper trapezius trigger points. Pak J Med Health Sci. 2023;17:873.

[24] Kashyap R Iqbal A Alghadir AH. Controlled intervention to compare the efficacies of manual pressure release and the muscle energy technique for treating mechanical neck pain due to upper trapezius trigger points. J Pain Res. 2018;11:3151–60. 30588067 10.2147/JPR.S172711 PMC6296190

[25] Alghadir AH Iqbal A Anwer S. Efficacy of combination therapies on neck pain and muscle tenderness in male patients with upper trapezius active myofascial trigger points. Biomed Res Int. 2020;2020:9361405. 32258159 10.1155/2020/9361405 PMC7085833

[26] Iqbal A Khan SA Miraj M. Efficacy of ischaemic compression technique in combination with strain counterstrain technique in managing upper trapezius myofascial trigger point pain. Indian J Physiother Occup Ther. 2010;4:10–5.

[27] Khan A Khan AR Zafar M. Efficacy of ischemic compression techniques and home exercise programme in combination with US among computer users with upper trapezius myofascial pain. Int J Health Sci Res. 2020;10:62–7.

[28] Alghadir AH Anwer S Iqbal A. Test–retest reliability, validity, and minimum detectable change of visual analog, numerical rating, and verbal rating scales for measurement of osteoarthritic knee pain. J Pain Res. 2018;11:851–6. 29731662 10.2147/JPR.S158847 PMC5927184

[29] Vernon H Mior S. “The neck disability index: a study of reliability and validity”. J Manipulative Physiol Ther. 1991;14:409–15. 1834753

[30] Jensen MP Karoly P Braver S. The measurement of clinical pain intensity: a comparison of six methods. Pain. 1986;27:117–26. 3785962 10.1016/0304-3959(86)90228-9

[31] Sawyer PC Uhl TL Mattacola CG. Effects of moist heat on hamstring flexibility and muscle temperature. J Strength Cond Res. 2003;17:285–90. 12741864 10.1519/1533-4287(2003)017<0285:eomhoh>2.0.co;2

[32] Hosoda T Kipshidze G Tsvid G. Type-I GaSb-based laser diodes operating in 31to 33$\mu $ m wavelength range. IEEE Photonics Technol Lett. 2010;22:718–20.

[33] Lari AY Okhovatian F Sadat Naimi S. The effect of the combination of dry needling and MET on latent trigger point upper trapezius in females. Man Ther. 2016;21:204–9. 26304789 10.1016/j.math.2015.08.004

[34] Doraisamy MA Anshul. Effect of latent myofascial trigger points on strength measurements of the upper trapezius: a case-controlled trial. Physiother Can. 2011;63:405–9. 22942517 10.3138/ptc.2010-27 PMC3207978

[35] Beckerman H de Bie RA Bouter LM. The efficacy of laser therapy for musculoskeletal and skin disorders: a criteria-based meta-analysis of randomized clinical trials. Phys Ther. 1992;72:483–91. 1409881 10.1093/ptj/72.7.483

[36] Guo Y Lv X Zhou Y. Myofascial release for the treatment of pain and dysfunction in patients with chronic mechanical neck pain: Systematic review and meta-analysis of randomised controlled trials. Clin Rehabil. 2023;37:478–93. 36305079 10.1177/02692155221136108

[37] Ahmed H Jarrar MA Ahmed R. Effect of post-isometric relaxation and laser on upper trapezius trigger point pain in patients with mechanical neck pain. Niger J Clin Pract. 2020;23:1660–6. 33355818 10.4103/njcp.njcp_6_20

[38] Junaid M Yaqoob I Shakil Ur Rehman S. Effects of post-isometric relaxation, myofascial trigger point release and routine physical therapy in management of acute mechanical neck pain: a randomized controlled trial. J Pak Med Assoc. 2020;70:1688–92. 33159734 10.5455/JPMA.15939

[39] Blanco CR de lasPeñas CF Xumet JEH. Changes in active mouth opening following a single treatment of latent myofascial trigger points in the masseter muscle involving post-isometric relaxation or strain/counterstrain. J Bodyw Mov Ther. 2005;10:197–205.

[40] Kay TM Gross A Goldsmith CH. Exercises for mechanical neck disorders. Cochrane Database Syst Rev. 2012. 10.1002/14651858.CD004250.pub4 22895940

[41] Vernon H Humphreys K Hagino C. Chronic mechanical neck pain in adults treated by manual therapy: a systematic review of change scores in randomized clinical trials. J Manipulative Physiol Ther. 2007;30:215–27. 17416276 10.1016/j.jmpt.2007.01.014

[42] Coskun Benlidayi I Guzel R Tatli U. The relationship between neck pain and cervical alignment in patients with temporomandibular disorders. Cranio. 2020;38:174–9. 30048225 10.1080/08869634.2018.1498181

[43] Balthillaya GM Parsekar SS Gangavelli R. Effectiveness of posture-correction interventions for mechanical neck pain and posture among people with forward head posture: protocol for a systematic review. BMJ Open. 2022;12:e054691. 10.1136/bmjopen-2021-054691 PMC8915312 35264350

